# Insights into IGH clonal evolution in BCP-ALL: frequency, mechanisms, associations, and diagnostic implications

**DOI:** 10.3389/fimmu.2023.1125017

**Published:** 2023-04-18

**Authors:** Franziska Darzentas, Monika Szczepanowski, Michaela Kotrová, Alina Hartmann, Thomas Beder, Nicola Gökbuget, Stefan Schwartz, Lorenz Bastian, Claudia Dorothea Baldus, Karol Pál, Nikos Darzentas, Monika Brüggemann

**Affiliations:** ^1^Medical Department II, Hematology and Oncology, University Hospital Schleswig-Holstein, Kiel, Germany; ^2^University Cancer Center Schleswig-Holstein (UCCSH), University Hospital Schleswig-Holstein, Kiel, Germany; ^3^Clinical Research Unit “CATCH-ALL” (KFO 5010/1), funded by the Deutsche Forschungsgemeinschaft (DFG, German Research Foundation), Bonn, Germany; ^4^Department of Medicine II, Hematology/Oncology, Goethe University Hospital, Frankfurt/M, Germany; ^5^Department of Hematology, Oncology and Tumor Immunology, Charité - Universitätsmedizin Berlin, corporate member of Freie Universität Berlin and Humboldt-Universität zu Berlin, Berlin, Germany; ^6^German Cancer Consortium (DKTK), German Cancer Research Center (DKFZ), Heidelberg, Germany; ^7^Central European Institute of Technology, Masaryk University, Brno, Czechia

**Keywords:** acute lymphoblastic leukemia, clonal evolution, minimal residual disease, DNJ-stem, V_H_ replacement, IGH rearrangements, high-throughput sequencing

## Abstract

**Introduction:**

The malignant transformation leading to a maturation arrest in B-cell precursor acute lymphoblastic leukemia (BCP-ALL) occurs early in B-cell development, in a pro-B or pre-B cell, when somatic recombination of variable (V), diversity (D), and joining (J) segment immunoglobulin (IG) genes and the B-cell rescue mechanism of V_H_ replacement might be ongoing or fully active, driving clonal evolution. In this study of newly diagnosed BCP-ALL, we sought to understand the mechanistic details of oligoclonal composition of the leukemia at diagnosis, clonal evolution during follow-up, and clonal distribution in different hematopoietic compartments.

**Methods:**

Utilizing high-throughput sequencing assays and bespoke bioinformatics we identified BCP-ALL-derived clonally-related IGH sequences by their shared ‘DNJ-stem’.

**Results:**

We introduce the concept of ‘marker DNJ-stem’ to cover the entirety of, even lowly abundant, clonally-related family members. In a cohort of 280 adult patients with BCP-ALL, IGH clonal evolution at diagnosis was identified in one-third of patients. The phenomenon was linked to contemporaneous recombinant and editing activity driven by aberrant ongoing D_H_/V_H_-DJ_H_ recombination and V_H_ replacement, and we share insights and examples for both. Furthermore, in a subset of 167 patients with molecular subtype allocation, high prevalence and high degree of clonal evolution driven by ongoing D_H_/V_H_-DJ_H_ recombination were associated with the presence of *KMT2A* gene rearrangements, while V_H_ replacements occurred more frequently in Ph-like and DUX4 BCP-ALL. Analysis of 46 matched diagnostic bone marrow and peripheral blood samples showed a comparable clonal and clonotypic distribution in both hematopoietic compartments, but the clonotypic composition markedly changed in longitudinal follow-up analysis in select cases. Thus, finally, we present cases where the specific dynamics of clonal evolution have implications for both the initial marker identification and the MRD monitoring in follow-up samples.

**Discussion:**

Consequently, we suggest to follow the marker DNJ-stem (capturing all family members) rather than specific clonotypes as the MRD target, as well as to follow both VDJ_H_ and DJ_H_ family members since their respective kinetics are not always parallel. Our study further highlights the intricacy, importance, and present and future challenges of IGH clonal evolution in BCP-ALL.

## 1 Introduction

The immense diversity of the antibody repertoire in humans is physiologically conveyed by mechanistic editing and assembly of immunoglobulin (IG) genes, particularly in the processes of somatic recombination of variable (V), diversity (D), and joining (J) segment genes. In the early developmental stage of a pro-B cell, one D_H_ and one J_H_ gene segment of the IG heavy (H) chain locus become joint, eventually followed by V_H_ to DJ_H_ recombination. Consequently, the somatic VDJ_H_ recombination yields a uniquely rearranged IGH DNA sequence of the VDJ_H_ joining region in each B lymphocyte. The joining is an imprecise process, in which germline segment ends are cleaved by the RAG1/2 recombinase activity and joined upon random addition of “non-templated” nucleotides (N nucleotides) into the junctions between segments. The resulting DNA sequence variations add to the overall diversity of antigen receptors and constitute a unique “fingerprint” target for the design of leukemia-clone specific MRD markers. They also offer a valuable tool to decipher which mechanism of somatic recombination was active and to gather clonally related clonotypes (1–4).

Malignant transformation is preceded by a premalignant state, in which genetic aberrations accumulate over time until the driver lesion finally complements the transformation process. Expanded malignant cells harbor the successively acquired genetic aberrations allowing for backtracking the mutational trajectories and (sub)clonal architecture of the malignant population. In the case of malignantly transformed immune cells such as B and T lymphocytes, these carry the unique VDJ rearrangement of the preleukemic cell of origin. Importantly, the VDJ recombination is a stepwise and temporarily tightly regulated process, thus, the completeness of the rearrangements basically depends on the developmental state of the malignantly transformed cell of origin.

The malignant transformation in B-cell precursor acute lymphoblastic leukemia (BCP-ALL) occurs at an early stage in the B-cell development, hitting precursor B cells in the stage of a pro-B or a pre-B cell. Therefore, the process of somatic VDJ recombination might still be active in the malignant B cell, driving IGH clonal evolution either through recombination of incomplete DJ_H_ rearrangements with multiple V_H_ segments (occasionally with D_H_ segments as D_H_-D_H_ tandem fusion rearrangements) or by the process of V_H_ replacement (5–11).

The aforementioned processes are mediated by the RAG1/2 recombinase activity, which recognizes the canonical recombination signal sequences (RSS) during V_H_-DJ_H_ recombination as well as cryptic RSS during D_H_-DJ_H_ recombination and V_H_ replacement (10, 12, 13). In the latter process, the cryptic RSS of the original V_H_ segment and the conventional RSS of the incoming V_H_ segment are processed to replace the original V_H_ segment by the incoming with the original DJ_H_ assembly kept. V_H_ replacement, which can go on for multiple rounds, may leave a remnant of up to five base pairs of the original V_H_ gene, which can be further diversified by exonuclease activity or N nucleotide addition. V_H_ replacement also occurs in healthy humans in bone marrow immature B cells to rescue B cells with nonfunctional or autoreactive receptors (14–16).

IGH clonal evolution in BCP-ALL might pose a serious diagnostic challenge for IGH-based MRD assessment upon further diversification of leukemic clonotypes during long-term follow-up. MRD monitoring utilizing clone-specific sequences of the uniquely rearranged and edited gene segments is widely used to assess therapy response after induction and consolidation therapy, which has crucial prognostic importance in BCP-ALL patients (17–23). High-throughput sequencing (HTS)-based marker identification and MRD monitoring offers the benefit of providing additional information on background repertoire including minor accompanying clones, which may reflect clonal evolution [reviewed in (24)].

In this study of BCP-ALL diagnostic and follow-up bone marrow (BM) and peripheral blood (PB) materials utilizing HTS techniques to backtrack rearranged leukemic IGH sequences and their trajectories, we sought to understand the mechanistic details of leukemic clonal composition at diagnosis, clonal evolution during follow-up, and clonal distribution in different body or hematopoietic compartments. We link diversified IGH clonal family members and discriminate between the mechanisms driving clonal evolution. We correlate these results with immunophenotypes obtained by multiparameter flow cytometry and with transcriptome-based molecular subtypes. We report on the frequency of the phenomenon of clonal evolution, provide examples of clonal sustainability under immunotherapy and finally discuss the implications of clonal evolution in BCP-ALL with regard to initial diagnostics and MRD monitoring.

## 2 Methods

### 2.1 Patients and clinical samples

**Figure 1:**
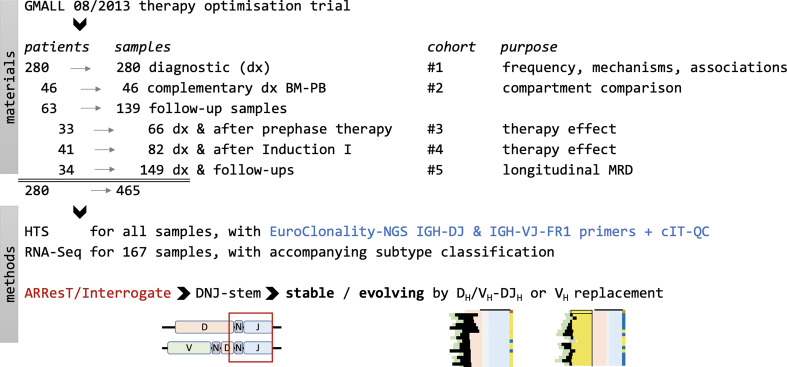
Study overview. A total of 465 samples from 280 patients with BCP-ALL enrolled in the GMALL 08/2013 therapy optimization trial were analyzed in five different cohorts to decipher frequency, mechanisms and kinetics of the leukemic clonal evolution. The bioinformatic analyses on IGH HTS data were carried out on DNJ-stem sequences identified by ARResT/Interrogate (25). Transcriptome sequencing and molecular subtype calling were performed as described by Bastian et al. (26) HTS, high-throughput sequencing; RNA-Seq, RNA next- generation sequencing.

A total of 465 diagnostic and selected MRD-positive follow-up samples from 280 patients aged 18-55 years with BCP-ALL were investigated within the frame of a research project associated with the German Multicenter Adult Acute Lymphoblastic Leukemia (GMALL) 08/2013 therapy optimization trial (EudraCT-No.: 2013-003466-13). Initial diagnostic samples were subjected to IGH amplicon-based HTS, multiparameter flow cytometry, and transcriptome sequencing (RNA-Seq) analyses. An overview of patient characteristics is detailed in **Supplementary Table 1**. From all 280 patients either diagnostic BM (n=217) or PB (n=63) samples were available (**Figure 1**, cohort #1). Concurrent diagnostic PB and BM samples were available for pairwise comparisons in 46/280 cases (**Figure 1**, cohort #2). In addition, a total of n=139 MRD-positive early or late follow-up and relapse samples of 63 patients were selected. Clonal IGH evolution was monitored during early therapy phases, in particular during the cyclophosphamide/dexamethasone-containing prephase (**Figure 1**, cohort #3, 66 paired samples from 33 patients) and after Induction I including one dose of rituximab (**Figure 1**, cohort #4, 82 paired samples from 41 patients). Ultimately, we studied 149 diagnostic and follow-up samples of 34 patients to track the kinetics of clonal evolution over time in longitudinal case reports (**Figure 1**, cohort #5). Main results and details on IGH sequences within the cohorts #1-5 are summarized in **Supplementary Table 4**. All patients provided informed consent. All research described herein was approved by the Frankfurt Research Ethics Board (188/15F) and performed in accordance with the Declaration of Helsinki.

### 2.2 Routine diagnostics

Initial diagnoses were established by standard routine diagnostics. Initial immunophenotype, *BCR::ABL1* and *KMT2A* rearrangement status, initial molecular IG/TR markers, and MRD at follow-up were obtained by GMALL trials central diagnostic reference laboratories (Berlin & Kiel, Germany).

### 2.3 Transcriptome analysis

RNA was extracted according to standard procedures recommended by the manufacturer (Trizol, Life Technologies, Carlsbad, CA). Library preps, transcriptome sequencing, and molecular subtype calling were performed as described previously (26). Cases with intermediate or divergent gene expression profiles could not be assigned to any existing molecular profile category and were thus described as “other”.

### 2.4 High-throughput sequencing of the IGH locus

We employed the EuroClonality-NGS assay and the IGH-VJ-FR1 and IGH-DJ primer sets to sequence diagnostic and follow-up samples. In general, 100 ng of DNA, extracted according to standard procedures recommended by the manufacturer (AllPrep, Qiagen, Hilden, Germany), was used for the analysis of the diagnostic samples. In the longitudinal analysis of diagnostic and follow-up samples, we used 500 ng DNA to ensure adequate sensitivity and direct comparability of results. The EuroClonality-NGS central in-tube quality/quantification control (cIT-QC) was spiked into most diagnostic samples and all follow-up samples as a library-specific quality control and for the normalization of abundance from reads to cells (25, 27, 28). (www.euroclonality.org/protocols). Samples were sequenced on a MiSeq (Illumina, San Diego, CA, USA) with 2x250bp reads (sequencing depth analysis in **Supplementary Material S1**).

### 2.5 IGH sequence analysis, detection, and characterization of clonal evolution

IGH sequences were analyzed with ARResT/Interrogate (25). Usable reads, the denominator for percentage abundance calculations, were defined as sample reads with identified junctions after the exclusion of cIT-QC reads, in other words, reads with patient-only IGH rearrangements. For better readability, especially in figures, we will refer to IGH segment rearrangements using name acronyms omitting ‘IGH’ from gene names, e.g., V_H_ instead of IGHV or DJ_H_ instead of IGHD-IGHJ.

**Figure 2:**
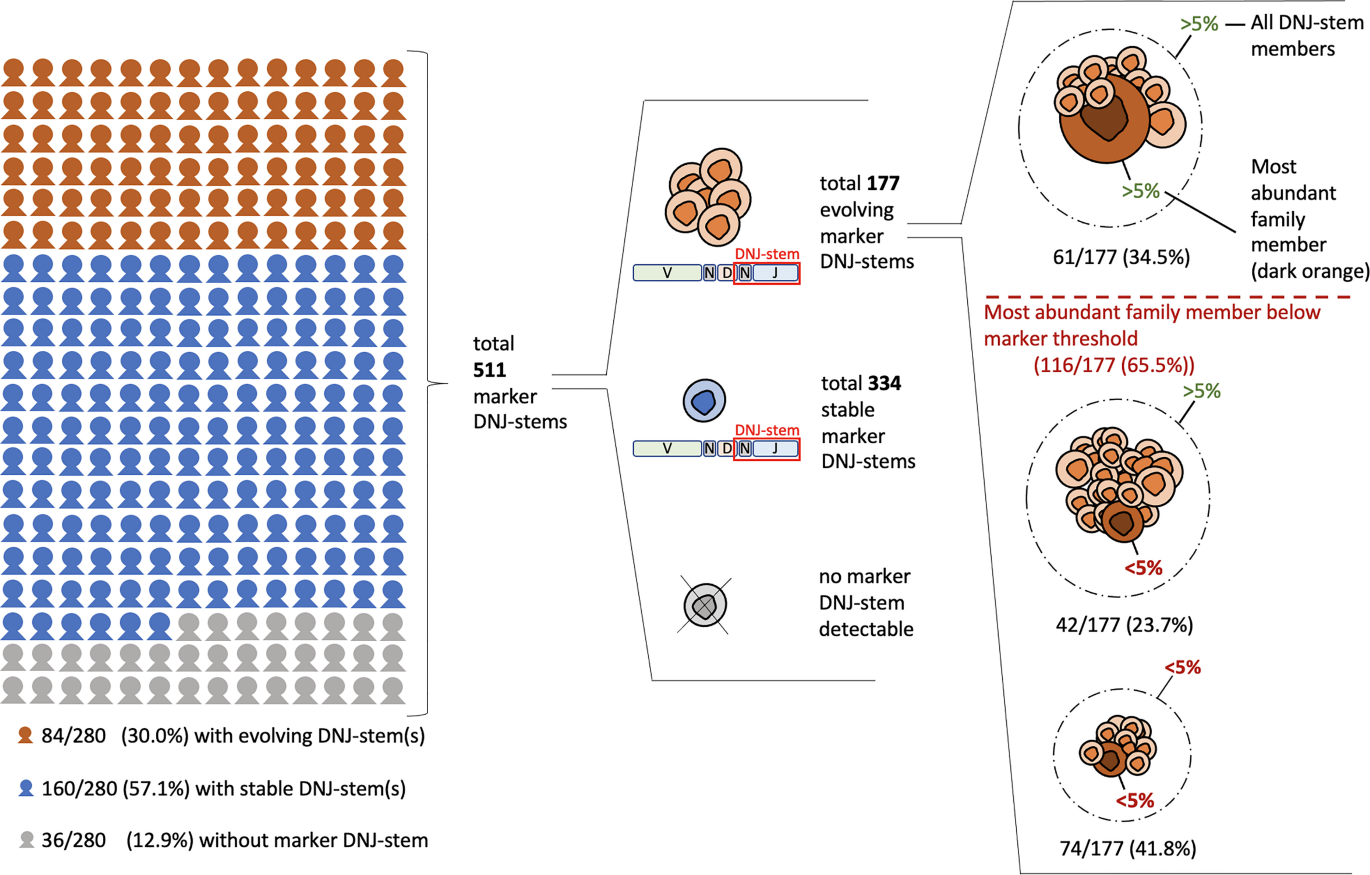
Clonal evolution and clonotypic abundances in diagnostic BCP-ALL samples (cohort #1). The figure is displaying the prevalence of clonal evolution in reference to all 280 patients and in reference to the total of 511 detected marker DNJ-stems. Additionally, the evolving DNJ-stems are itemized by their most abundant family member abundance and total DNJ-stem abundance relating to the 5% marker threshold.

For IGH clonal evolution assessment, we isolated the ‘DNJ-stem’ of nucleotide junctions - a subsequence of the junction that consists of the last maximum of 3 D_H_ nucleotides (or if none are identifiable in a VJ_H_ rearrangement, of the N region), any N nucleotides between D_H_ and J_H_, and all J_H_ nucleotides (**Figure 2**). The DNJ-stem definition is adapted to the understanding that the corresponding junction subsequence is the one remaining generally stable after multiple D_H_/V_H_ to DJ_H_ (D_H_/V_H_-DJ_H_) recombination and V_H_ replacement events, and it is therefore used as the link, a shared denominator, between potential family members – whether those clonotypes should indeed be considered related and whether the DNJ-stem should be considered evolving was thoroughly examined by deploying further rules.

We initially removed - as potential noise - clonotypes highly similar to the most abundant DNJ-stem family member, as well as of abundance below 3 reads or below 0.01% of usable reads. We then considered the number of clonotypes removed above (a high number may indicate an overall noisy DNJ-stem), the absolute and relative (to the sample total) number of remaining DNJ-stem family members, the number of their different 5’ genes and junction lengths as a sign of expected junctional variability, as well as the DNJ-stem’s N region complexity to increase confidence in its specificity. Combined consideration of all these data points led to a consensus call on whether a DNJ-stem was evolving or stable. Please see **Supplementary Material S2** for the detailed pseudocode of this algorithm.

V_H_ replacement was identified by considering (i) the general stability of the D_H_ region’s 5’-site and its preceding N1 region’s 3’-site and (ii) the presence of a sequence remnant (fingerprint) of the replaced V_H_ in the new junction. Evolving DNJ-stems with the absence of aforementioned signs were categorized as D_H_/V_H_-DJ_H_ recombinations.

If a DNJ-stem was detected in both the IGH-VJ and IGH-DJ library, the respective DNJ -stem was termed ‘rooted’, indicating that the underlying root DJ_H_ rearrangement was also detectable.

A marker DNJ-stem was defined if any of the following conditions were fulfilled: (i) abundance as a sum of all DNJ-stem family members ≥ 5% of usable reads or ≥ 1% of cells (normalized abundance); (ii) presence of clonal evolution (‘evolving’) at any abundance. We recovered the locus IGHV and IGHD gene order from IMGT (29).

Statistical analyses were performed in R version 4.1 using Fisher’s exact test with default settings, meaning the significance threshold was set at 0.05.

## 3 Results

### 3.1 Clonal evolution is frequent in BCP-ALL

We studied DNJ-stems and the respective VDJ_H_ and DJ_H_ rearrangements in the diagnostic cohort of 280 BCP-ALL patients (**Figure 1**, cohort #1). DNJ-stems with only DJ_H_ rearrangements were further analyzed only if evolving or otherwise noteworthy.

Clonal evolution, i.e. at least one evolving DNJ-stem, was found in 84/280 (30%) patients. Stable (non-evolving) DNJ-stems were found in 160/280 (57.1%) patients and 36/280 (12.9%) patients had no detectable IGH rearrangements to constitute a marker DNJ-stem – the latter being a rate comparable to previous studies (30, 31). In summary, clonal evolution was prevalent in our cohort affecting one-third of the cases (**Figure 2**).

In total, 511 marker DNJ-stems (i.e., of ≥ 5% usable reads or evolving at any abundance) were identified in 244/280 (87.1%) patients (range 1-11, average 2.1 per patient). In 116 (65.5%) out of the overall detected 177 evolving DNJ-stems, their most abundant DNJ-stem family member stayed below the generally accepted marker threshold of 5% usable reads and therefore may have been ignored on its own. In other words, applying the standard marker screening approach (by following single clonotypes and not DNJ-stems and using the conventional 5% threshold), 65.5% of markers would not have been reported. By screening for DNJ-stem instead of clonotypes, but still applying the 5% threshold, two-thirds (74) of those 65.5% of markers would still not have been reported. A visual breakdown of the DNJ-stems and family member distribution is depicted in **Figure 2**.

### 3.2 Leukemic clonal evolution is driven by ongoing D_H_/V_H_-DJ_H_ recombination and V_H_ replacements

**Figure 3:**
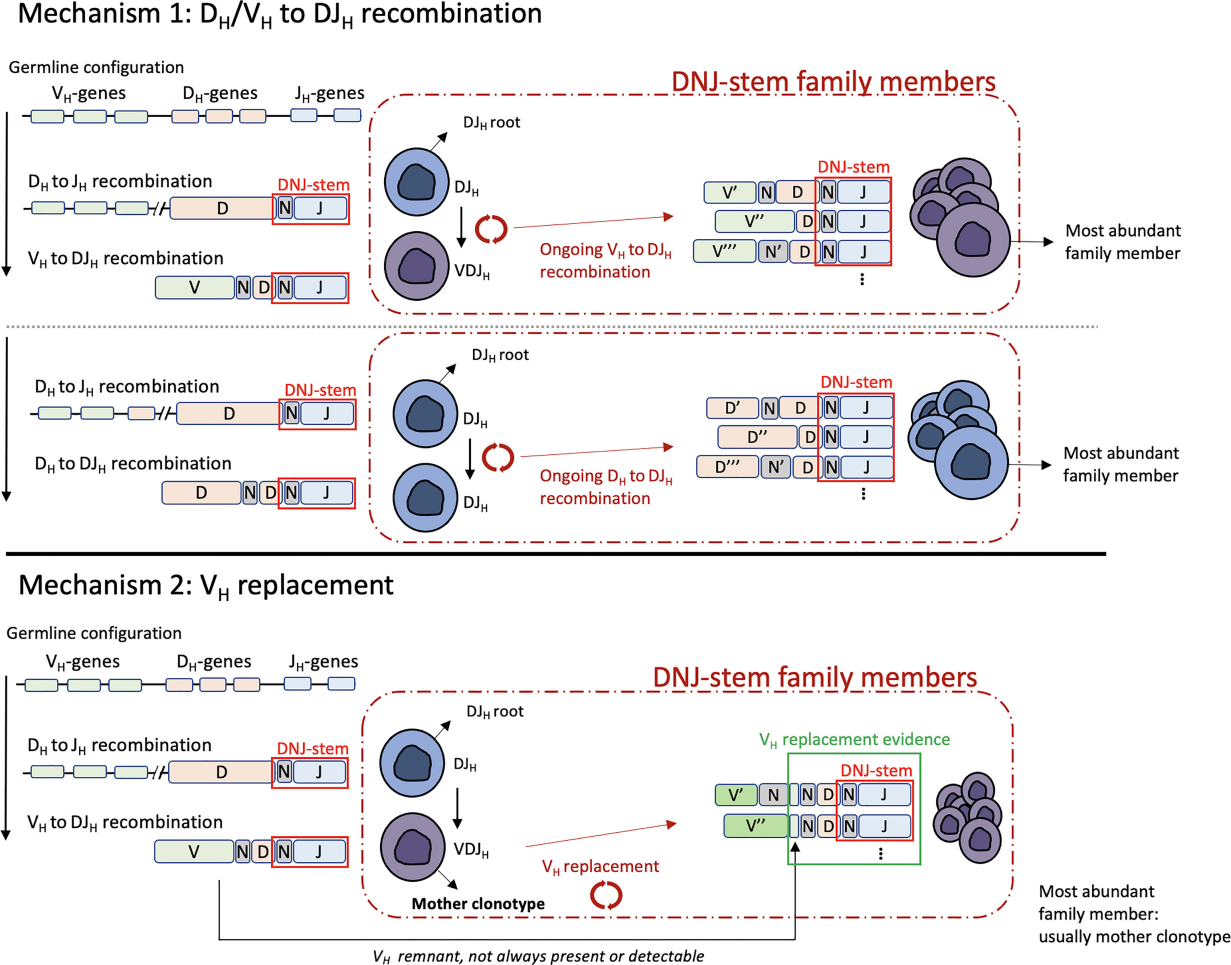
Schematic visualization of the terminology used in this study and the two mechanisms driving clonal evolution: D_H_/V_H_ to DJ_H_ recombination (top) and V_H_ replacement (bottom). All DNJ-stem family members, including the DJ_H_ root, the VJ_H_ mother clonotype (V_H_ replacement) carry the same DNJ sequence (red box). Evidence of V_H_ replacement (see main text) is not always present or detectable due to further recombination-related nucleotide sequence trimming (green box).

We studied alignments of junction nucleotide sequences of evolving DNJ-stem families from the diagnostic BCP-ALL samples (**Figure 1**, cohort #1) and observed signs of the two main mechanisms driving IGH clonal evolution, V_H_ to DJ_H_ (V_H_-DJ_H_) recombinations and V_H_ replacements. Additionally, we identified a variant of somatic recombination, in which D_H_ to DJ_H_ (D_H_-DJ_H_) recombination was evident (10, 11). Both mechanisms, D_H_/V_H_-DJ_H_ recombination and V_H_ replacement, leave distinctive changes in the recombined nucleotide sequences, by which they can be specifically discriminated (**Figure 3**).

**Figure 4:**
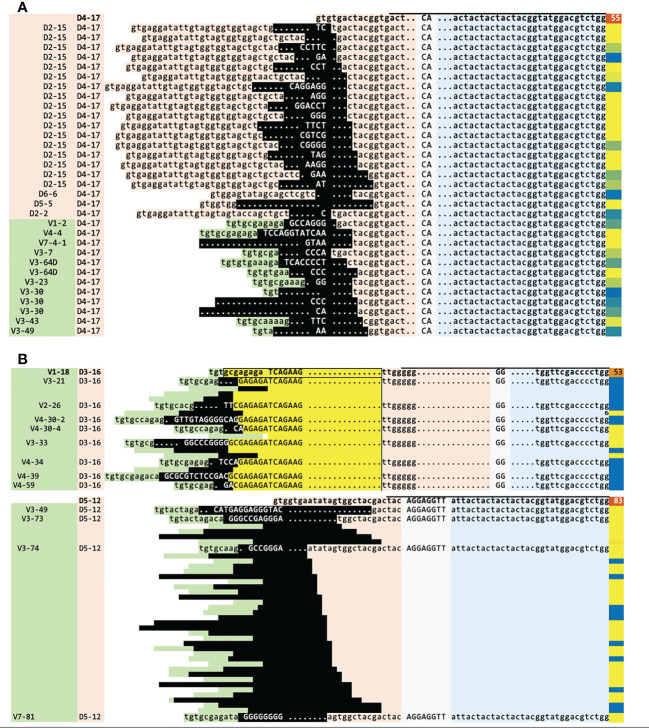
Sequence analysis of two cases illustrating the IGH clonal evolution mechanisms. **(A)** Case #61, ongoing D_H_-DJ_H_ and V_H_-DJ_H_ recombination on the same highly abundant DJ_H_ root (55% of reads) (top row, in bold). **(B)** Case #126, one of two unique cases with DNJ-stems featuring both mechanisms, V_H_ replacement and V_H_-DJ_H_ recombination (V_H_ replacement: top, V_H_-DJ_H_ recombination: bottom). The mother clonotype of the V_H_ replacement and the DJ_H_ root of the V_H_-DJ_H_ recombination are each shown on the top row and in bold. The DNJ-stem family members evolving by V_H_-DJ_H_ use ‘late’ D-distal V_H_ genes, which is uncommon for this mechanism (main text, **Figure 5**). Legend: green = V_H_, orange = D_H_, blue = J_H_, gray/uppercase = N regions, lowercase = germline, dots = deleted nucleotides, black = region affected by the recombination or replacement, yellow = region kept from the mother clonotype (on which the region is boxed), blue/yellow-to-orange (last column) = clonotype abundance in % reads, horizontal bar on top of first sequence = DNJ-stem. Genes are indented in the 1st column to indicate locus order. A number of rows in **(B)** are ‘squeezed’ and their V_H_ and D_H_ assignment and nucleotide sequences are hidden for space and clarity.

Ongoing D_H_/V_H_-DJ_H_ recombination was observed in 78% of evolving DNJ-stems. This mechanism results in sequence trimming during the rearrangement of the D_H_/V_H_ to the existing DJ_H_, which in addition to the random editing of N nucleotides results in sequence variability outside the DNJ-stem. This is exemplified in **Figure 4A**, case #61 and **Figure 4B**, lower part, case #126. In the illustration, the top row is the ‘mother’ clonotype for the family of clonotypes to be produced by parallel D_H_-DJ_H_ and V_H_-DJ_H_ recombination (case #61) or solely by V_H_-DJ_H_ recombination (case #126).

The vast majority of D_H_/V_H_-DJ_H_ cases featured only V_H_-DJ_H_ recombination-driven clonal evolution, but there were notable exceptions. In particular, of 398 DJ_H_ marker DNJ-stems identified in 144/280 (51.4%) patients, 21/398 (5.2%) DNJ-stems were evolving in 15 patients - in 12/15 (80.0%) of these patients this ongoing D_H_-DJ_H_ recombination accompanied the V_H_-DJ_H_ recombination, both driving clonal evolution (**Figures 4A, B**). The mechanism of V_H_ replacement was not active in those cases. Of note, the only two patients with D_H_-DJ_H_ recombination without accompanying V_H_-DJ_H_ recombination were assigned to the rare molecular subtype CDX2/UBTF, a novel BCP-ALL subgroup with the need of intensified treatment that was identified in 7% of our cohort (26) (see below).

Ongoing V_H_ replacement was observed in 22% of evolving DNJ-stems. T his mechanism is defined by its most distinct signs: (i) the general stability of the 5´-D_H_ region site and its preceding 3´-N1 region site; (ii) the actual nucleotide remnants of the replaced V_H_ in the new junction. There were very few exceptions to the requirement of the incoming V_H_ to be downstream from the mother clonotype segment: 1.6 upstream segments on average (range 1-5) in lowly abundant clonotypes, in 13/39 DNJ-stems evolving by V_H_ replacement, representing 1.9% of all family members. These could be erroneous gene annotations facilitated by sequence errors, false positive DNJ-stem members, or perhaps true members having originated previously. A case with ongoing V_H_ replacement is detailed in **Figure 4B** (upper part, case #126), where the top row is the ‘mother’ clonotype for the family members to be produced by replacement of its V_H_ segment. The resulting family members underneath show no further deletions in the D_H_ segment, because any sequence loss was buffered by the N1 region and remnants of the replaced V_H_ (highlighted in yellow) and now residing inside the new N1 regions.

Overall, and in contrast to V_H_ replacement that most commonly takes place in the presence of one or more highly abundant mother clonotypes, ongoing V_H_-DJ_H_ recombination results in bursts of numerous lowly abundant family members with the role of the mother clonotype taken by the DJ_H_ root. This translates to an average abundance of the most abundant family member of 51% for V_H_ replacement and 7% for V_H_-DJ_H_ recombination in the IGH-VJ library of our diagnostic cohort.

### 3.3 Clonal evolution associates with BCP-ALL immunophenotypes and molecular subtypes

**Figure 5:**
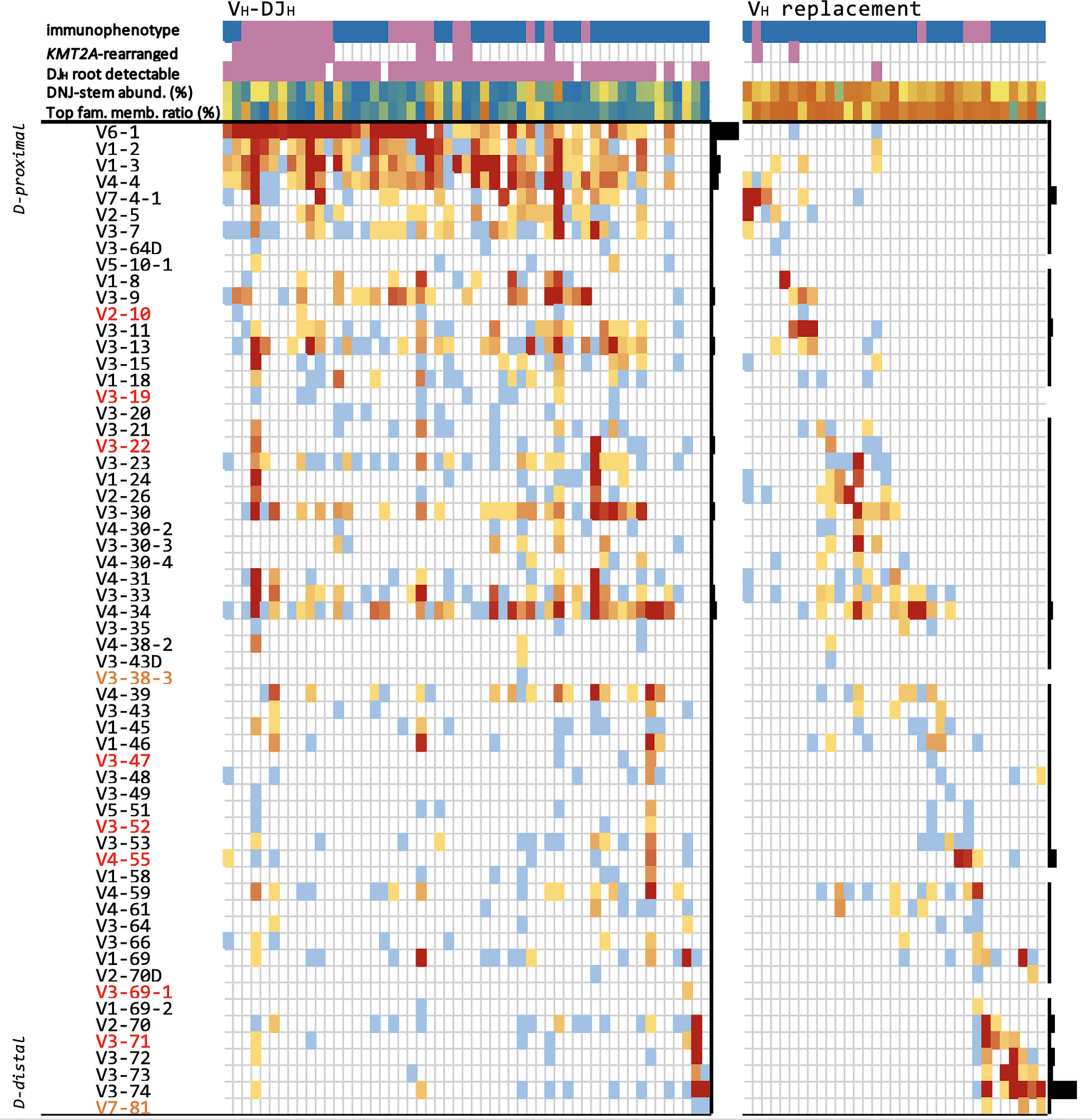
Evolving DNJ-stems in the diagnostic cohort. Each column represents one representative evolving DNJ-stem in one patient, ordered by the locus order of the most frequent gene in that column. V_H_ genes are sorted by their proximity to the D_H_ genes (proximal top to distal bottom), respectively; red gene names: pseudogenes, yellow: ORFs. Top rows: 1) immunophenotype: pro-B ALL purple, common-/pre-B ALL blue; 2) KMT2A molecular subtype status: KMT2Ar purple, non-KMT2Ar no color; 3) DJ_H_ root with same DNJ-stem detectable (purple); 4) DNJ-stem abundance (% reads), blue to orange (0.2% to 97%); 5) most abundant family member clonotype to summed DNJ-stem abundance ratio, blue to orange (0.01 to 0.99). Heatmap cells are colored by number of DNJ-stem family members featuring each gene, blue to red (1 to 418); black bar plots on the right of each mechanism group visualize each gene’s relative frequency in that group.

We selected one DNJ-stem from each patient or two DNJ-stems from each of two patients with concomitant V_H_-DJ_H_ recombination and V_H_ replacement - a total of 53 V_H_-DJ_H_-evolving stems and 33 V_H_ replacement-evolving stems - focusing on evolving DNJ-stems with numerous family members, to highlight specific characteristics related to both mechanisms driving clonal evolution. For each DNJ‐stem, abundances and IGH evolution classifications such as evolving or stable, DJ_H_ root detectable or not, D_H_/V_H_-DJ_H_ recombination or V_H_ replacement were annotated along with its locus-ordered V_H_ gene profile and basic patient metadata. The results are summarized in an overview tabular heatmap (**Figure 5**).

Of the 244 diagnostic samples of cohort #1 with identified marker DNJ-stems, 41 (16.8%) were classified as pro-B ALL and 203 (83.2%) as pre-B/c-ALL by multiparameter flow cytometry.

We detected significantly higher rates of clonal evolution in patients with a pro-B ALL compared to those with a pre-B/c-ALL immunophenotype (61.0% vs. 29.1%, respectively, *p*=0.0008), suggesting that the maturation arrest at earlier B-cell development stages might increase the frequency of ongoing IGH recombination.

Other noteworthy observations are that (i) V_H_-DJ_H_ overwhelmingly featured ‘early’, J-proximal V_H_ genes, mainly V_H_6-1 (42/53 (79.2%) DNJ-stems and most family members) - in strong contrast, V_H_6‐1 featured only twice in V_H_ replacement cases, in which the ‘late’, J-distal V_H_3-74 segment was the most frequent; (ii) the DJ_H_ root was present in the majority of V_H_-DJ_H_ DNJ-stems but in only one V_H_ replacement DNJ-stem; (iii) D_H_-DJ_H_-driven clonal evolution was observable in V_H_-DJ_H_ cases but not in V_H_ replacement cases; (iv) the two mechanisms, V_H_-DJ_H_ recombination and V_H_ replacement, were almost mutually exclusive (**Supplementary Tables 2, 3**).

**Figure 6:**
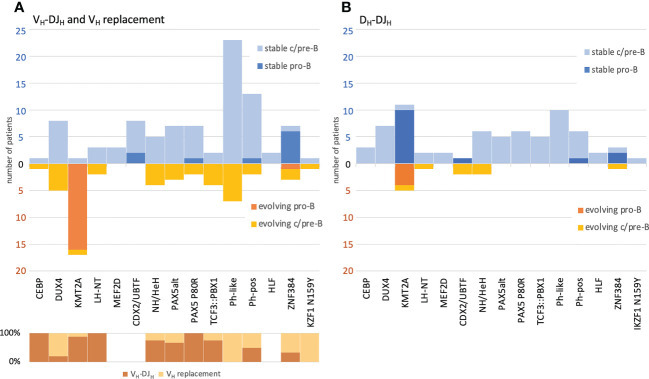
Correlation of BCP-ALL immunophenotype and molecular subtypes versus IGH clonal evolution and its respective mechanisms. VH replacement and VH-DJH recombination **(A)** and DH-DJH recombination **(B)**. The numbers and classifications are derived from the one-stem-per-case data depicted also in **Figure 5**. All cases have been labelled by (i) immunophenotype and (ii) their respective molecular subtype as assigned by the transcriptome analysis. Evolving DNJ-stems (warm colors) are shown below the X-axis line and stable DNJ-stems (cold colors) are shown above the X-axis line. For the evolving DNJ-stems, the respective mechanism driving clonal evolution in % of cases has been depicted in the lower left part of the schematic in **(A)**.

The BCP-ALL molecular subtypes were assigned based on RNA-Seq data available for 167/280 (59.6%) cases and then correlated with the IGH clonal evolution patterns. Overall, 155/167 cases could be assigned to a previously described molecular profile category, while 12 cases showed other gene expression profiles and thus were summarized as “other”. Notably, the aforementioned higher rates of clonal evolution in pro-B ALL were almost exclusively linked to the KMT2A subtype (94.4% evolving), while other molecular subtypes with a pro-B ALL immunophenotype were mostly stable, particularly CDX2/UBTF (cases with *UBTF::ATXN7L3* gene fusion, only 22.2% evolving and those only in the IGH-DJ library) and ZNF384 (only 30% evolving) (**Figure 6A**). Further, the KMT2A subtype showed significantly higher rates of ongoing V_H_-DJ_H_ (16/17 (94.1%) evolving patients, *p*=3e^‐11^), prevalently initiating the recombination with ‘early’ J-proximal V_H_ genes, mostly V_H_6-1 (**Figure 5**). The preferential usage of ‘early’ J-proximal V_H_ genes was also seen in the molecular subtypes PAX5 P80R and PAX5alt. Although (and in contrast to the KMT2A subtype) most DNJ‐stems in those subtypes were stable (30/40, 75%), the evolving DNJ-stems (10/40, 25%) clearly showed a prevalent (as in 7/10) usage of ‘early’ J-proximal V_H_ segment genes including V_H_6-1, V_H_1‐2, V_H_1-3 and V_H_4-4. Notably, the three DNJ-stems using more J-distal V_H_ segments belonged to a single patient with concurrent V_H_ replacement and V_H_-DJ_H_ recombination activity (**Supplementary Table 2**). While the KMT2A subtype was strongly associated with V_H_-DJ_H_ recombination as the prevalent clonal evolution mechanism, the molecular subtypes Ph-like and DUX4 featured no V_H_-DJ_H_ but were instead associated with V_H_ replacement (7/37 and 4/13, *p*=0.00009 and *p*=0.067, respectively) (**Figure 6A**).

The D_H_-DJ_H_ evolution was a less frequent observation and only detectable in 11 of 155 (7.1%) patients with molecular subtype assignment, with the low number of patients impeding significant associations with molecular subtypes. Despite the fact that the KMT2A subtype not only showed the highest rate of V_H_-DJ_H_ evolution, but also significantly higher rates of D_H_-DJ_H_ evolution (27.8% evolving, *p*=0.0001), we found that the presence of D_H_-DJ_H_ and V_H_-DJ_H_ was not fully concordant (**Figure 6B**). Strikingly, two patients of the rare CDX2/UBTF subtype showed only D_H_-DJ evolution (*p*=0.04), whereas there were no signs of V_H_-DJ_H_ evolution. Overall, the evolving D_H_-DJ_H_ rearrangements showed a remarkable preference for the usage of D_H_2-2 as the incoming D_H_ (14/15 (93.3%) of DNJ-stems) (**Supplementary Table 3**), which was also observed by others but in the context of normal rearrangements in blood B cells from healthy donors (32).

### 3.4 Overlap between BM and PB is high for marker DNJ-stems but not for their family members

**Figure 7:**
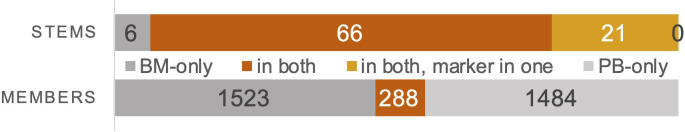
Overlap of marker DNJ-stems and their family members detectable in BM and PB. Total numbers of marker DNJ-stems (top) and DNJ-stem family members (bottom) that were found in only one of the compartments are illustrated in grey. Those that were overlapping between the compartments are illustrated in orange. In case of marker DNJ-stems, we distinguished between concordant marker status (dark orange) and detectable in both, but only marker status in one (light orange).

We sought to understand the overlap of DNJ-stems and their family members between diagnostic BM and PB of the 46 patients with both diagnostic materials available (**Figure 1**, cohort #2). We identified 39 patients with paired BM and PB DNA samples and with marker DNJ-stem(s) in at least one of these two compartments. The total number of DNJ-stems was 93, with 66 DNJ-stems in both compartments as marker DNJ-stems (see Methods for definition), 21 DNJ-stems also in both compartments but appearing in one compartment outside the marker DNJ-stem rules (and almost always at very low abundance), and the remaining 6 appearing only in BM compartment (**Figure 7**). This translated into 37/39 patients with at least one of the 87 DNJ-stems in both materials, or 36/39 when we demanded marker DNJ-stems in both materials, i.e., considering only the 66 DNJ-stems; all other cases had the DNJ-stem in BM only. Of the 66 DNJ-stems in both the BM and PB compartments of the 36 patients, all but two had concordant clonal evolution status between BM and PB, with the two exceptional cases evolving in BM and stable in PB. In 32/36 patients we saw a complete marker DNJ-stem overlap between BM and PB.

When we compared the overlap of ~3300 family members of 26 DNJ-stems that were evolving both in BM and PB, we found the overlap to be only 8.7%, with the rest split almost equally between BM and PB (**Figure 7**). Evolving DNJ-stem abundance in BM was on average 18.7% reads (range 0.6-89.9%), while the average abundance of family members was 0.16% reads (range 0.0008-71.8%). In PB the evolving DNJ-stem abundance was on average 12.9% reads (0.2-76.8%) and the average abundance of family members 0.11% (range 0.0002-73.8%). The fact that the minimum abundance of family members is below the 0.01% reads threshold used for family members when calling clonal evolution, is due to searching for members across materials at any abundance to maximize the sensitivity and information content of the overlap analysis.

### 3.5 Clonal evolution over time and after therapy – implications for MRD diagnostics

In order to understand the longitudinal dynamics of evolving clonotypes during and after treatment, we compared therapy-naive BCP-ALL samples (day 0) and matched samples from different follow-up time points (**Figure 1**, cohort #3-5): (i) day +6 samples after the 5-day cyclophosphamide/dexamethasone-containing prephase, no rituximab (cohort #3); (ii) day +22 samples after Induction I including one dose of rituximab (cohort #4); and (iii) later time points including additional doses of rituximab and refractory disease/relapse-related secondary immunotherapies, e.g. blinatumomab (cohort #5).

#### 3.5.1 The clonotypic spectrum does not change much during the very early phase of therapy

Cohort #3 encompassed 33 BCP-ALL patients with a total of 66 matched peripheral blood (PB) samples taken at diagnosis and at day +6 of the prephase. In 3/33 (9.1%) diagnostic PB samples, no marker DNJ-stem was identified. In one case, the stable marker DNJ-stem was undetectable after prephase therapy. More than two-thirds of the cases were either concordantly stable (19/33, 57.6%) or concordantly evolving (4/33, 12.1%) across day 0 and day +6. Four cases (12.1%) were evolving at day 0 and stable at day +6, with the loss of family members after prephase therapy probably due to the general reduction of tumor burden. Two cases showed clonal evolution after prephase therapy but not at diagnosis, which could be rather attributed to the overall reduction of benign mature B-cell numbers in the post-prephase samples and the resulting higher sensitivity of HTS. In the majority of concordantly evolving DNJ-stems (4/6), the highest abundant family member at day 0 remained the highest abundant family member at day +6. The overlap of evolving clonotypes between the two time points was on average 29.6%.

We further quantified the MRD at day +6 using either the DNJ-stem, or only the most abundant family member as MRD marker and compared the MRD levels. The DNJ-stem-based MRD was on average 3.5-times higher (range 1.1 to 9.7-times) than the most abundant family member-based MRD. In 2/8 evolving DNJ-stems, the discrepancy between DNJ-stem MRD and most abundant family member MRD equaled a log10 difference.

#### 3.5.2 Single dose rituximab and early Induction therapy unlikely to cause a clonotypic selection

Cohort #4 encompassed 41 BCP-ALL patients with a total of 82 matched bone marrow (BM) samples taken at diagnosis and at day +22 after Induction I with 1 dose of rituximab administered after the prephase. In 6/41 (14.6%) of initial diagnostic samples, no DNJ-stem was identified. The evolving marker DNJ-stem in one case and a stable marker DNJ-stem in another two cases were undetectable after Induction I. In accordance with cohort #1, nearly two-thirds of patients were either concordantly stable (20/41, 48.8%) or concordantly evolving (7/41, 17.1%), with 5 patients evolving at day 0 and stable after Induction I. However, no cases were stable at diagnosis but evolving after Induction I. The average overlap of evolving clonotypes was 28.5%.

#### 3.5.3 Clonotypes display more dynamic changes in longitudinal analysis

Cohort #5 encompassed 34 BCP-ALL patients with a total of 149 matched PB and/or BM samples taken at diagnosis and at multiple follow-up time points per patient, i.e. at the end of prephase at day +6, after Induction I at day +22, at later time points with refractory disease or/and at relapse during chemotherapy including 4 or 8 doses of rituximab or at relapse after additional immunotherapy with blinatumomab or inotuzumab ozogamicin. In particular, the clonotypic composition of initial diagnostic samples was compared to 97 matched samples obtained during these aforementioned divergent MRD-positive follow-up time points. In order to evaluate in how many cases the conventional MRD detection (which relies on the tracking of the one or two most abundant family members) would have been hampered by the vanishing of the most abundant family member, we analyzed clonal evolution dynamics over time in the 34 patients of cohort #5. At diagnosis 14 of 34 patients (41.2%) had stable DNJ-stems (no clonal evolution), 4 (11.8%) had no IGH marker and 16 (46.4%) were evolving. In those 16 patients with clonal evolution, we identified 45 evolving DNJ-stems in total (**Supplementary Table 4**). Noteworthy, 17/45 (37.8%) of evolving DNJ-stems including all family members became untraceable in the follow-up samples. In detail, 15/17 evolving DNJ-stems were distributed among two patients, in whom the MRD remained only traceable through the *KMT2A* fusion-derived MRD marker assay, underscoring the importance of this alternative approach of MRD monitoring in *KMT2A*-rearranged B-ALL. The remaining 2/17 disappearing evolving DNJ-stems were both related to one patient and presented the most abundant clonotypes at diagnosis but became eradicated during the follow-up. In 7/45 (15.6%) of evolving DNJ-stems, the most abundant family member stayed most abundant across all follow-up time points. In 2/45 (4.4%) evolving DNJ-stems the most abundant family member was traceable at all the time points, but did not stay the most abundant family member at all time points, thus not reflecting the most correct MRD level. In the majority of evolving DNJ-stems (19/45, 42.2%), the most abundant family member disappeared over time, while other family members of the same DNJ-stem remained present and would be available to constitute the MRD signal in the DNJ-stem based MRD approach. The median abundance of the most abundant family member in those 19 evolving DNJ-stems was 0.06% (0.002 -37.7%) cells compared to 20% (0.67 - 87.6%) cells in the 7 evolving DNJ-stems, in which the most abundant family member remained most abundant at later time points. The non-persistence of the most abundant family member was most likely caused by its low abundance in follow-up in combination with a sampling bias. In one case (**Supplementary Material S3A**, Ph-neg#27), a highly abundant family member (37.7% cells) disappeared in the course of therapy, while only the second most abundant family member persisted. Taken together, substantial changes in the abundance and composition of the initially most abundant family members conventionally chosen as molecular MRD markers were observed in 37/45 (82%) of the evolving DNJ-stems and accordingly in 9/16 (56%) patients with evolving DNJ-stems, in whom the correct assignment of the MRD level could be adversely affected, i.e. underestimated or failing detection. Critically, in 2/34 (5.9%) of patients, the DNJ-stem with the vanishing most abundant family member was the only IGH target for MRD detection.

In the **Supplementary Material S3A-D** we present four examples of Philadelphia chromosome-negative (Ph-neg) cases with interesting dynamics of family members belonging to the same DNJ-stem, all presenting different kinetics over time, in which the conventional marker identification (5% reads threshold) or the conventional MRD quantification (clonotype based) would have led to impaired MRD detection in follow-up samples.

Of note, in all cases, the mechanism of clonal evolution in all follow-up samples remained the same compared to the respective diagnostic sample. In consideration of the cohorts #3-5, we conclude that applying the following rules could improve MRD monitoring in follow-up samples: (i) consider the DNJ-stem and not specific family members as MRD basis marker, also because new family members of initially stable DNJ-stems could emerge during the course of therapy; (ii) if an evolving DNJ-stem in the IGH-VJ library is also found in the IGH-DJ library sample (and thus rooted), one should also follow the DNJ-stem in the IGH-DJ library for MRD monitoring even if it does not fulfill marker criteria in the diagnostic sample; (iii) use DNJ-stem abundance as the sum of all family members to avoid underestimation of the extent of MRD.

## 4 Discussion

Clonal evolution in the IGH locus and its significance and implications in BCP-ALL patients have all been well documented (5, 33–35). Our research was conducted on a well-studied cohort in order to develop tools for the identification and characterization of this important phenomenon. We provide refined observations with a more detailed insight into clonal evolution processes during BCP-ALL therapy.

We report that IGH clonal evolution is prevalent in BCP-ALL, conservatively identified in one-third of the cases. Clonal evolution has been shown to be a malignancy-related phenomenon of B cells (8). The approach presented in this manuscript to identify IGH clonal evolution and the resulting IGH oligoclonality is not based on the simple detection of more than two dominant clonal IGH rearrangements, which could be present even without oligoclonality in the case of biallelic rearrangements or trisomy of the IGH locus (e.g., in hyperdiploid ALL) (36, 37). Instead, we introduce the concept of ‘marker DNJ-stem’ to cover the entire, clonally-related family of evolving clonotypes. We identified 40% of evolving marker DNJ-stems at levels below conventional clonality thresholds (<5% reads) in 38/280 (14%) patients presenting clonal evolution. In such cases, conventional abundance thresholds produce misleading results and should therefore not be applied. In other words, evolving DNJ-stems at any level should be assumed to be BCP-ALL-related and therefore considered as a marker.

We further investigated whether the degree of clonal evolution and the composition of DNJ-stem family members differs between BM and PB. Comparing the diagnostic sample pairs of 46 patients, we found that the high overlap of marker DNJ-stems and their evolution status indicates that PB is a comparable and reliable source for the determination of the IGH oligoclonality status. The V_H_ usage of DNJ-stem family members did not show a tendency towards more mature clonotypes in PB compared to BM. In line with our previous study, which showed that MRD levels tend to be lower in PB than in BM (38), 22% of marker DNJ-stems were exclusively found in BM, where DNJ-stems and clonotypes are generally more abundant and evolving, indicating that not all malignant family members access the bloodstream in sufficient numbers to be detected. As expected, the combination of the low abundance of evolved family member clonotypes and sampling biases resulted in their low overlap between the two materials - but of note, their total number was not higher in BM compared to PB.

Abandoning the conventional 5% reads clonality threshold for evolving DNJ-stems as suggested above entails the risk of reporting unspecific, malignancy-unrelated markers which can result in impaired MRD detection. Thus, it should be discussed if the DNJ-stem is unique and specific enough to be a reliable MRD target. The insertion of short palindromic P nucleotides during VDJ_H_ recombination has been reported in around ten percent of IGH rearrangements (39). These nonrandom sequences might reduce the specificity of the DNJ-stem for MRD detection, especially in cases with short or completely absent N2 nucleotides. Furthermore, it is not clear if the application of DNJ-stem-based MRD detection is of benefit in mature lymphoid malignancies. We, therefore, tested the sensitivity and specificity of this approach using IGH sequences in internal cohorts of two distinct mature B-lymphoid malignancies, i.e. ~100 chronic lymphocytic leukemia (CLL) and ~100 multiple myeloma (MM) cases. While we detected no clonal evolution in the CLL cohort, three DNJ-stems in as many cases in the MM cohort were reported. All three were ‘borderline’ calls with few family members and low variability of genes and junction lengths, calls that in the context of MM could be dismissed. Additionally, we screened our BCP-ALL cohort for marker DNJ-stems that appear in more than one patient at diagnosis. Only one of the 511 marker DNJ-stems detected in the IGH-VJ library was shared between two patients. Overall, we would advise the application of bio/medical context during the interpretation of results, and to examine DNJ-stem sequences for specificity within each cohort and against a large reference cohort.

The study of the junction alignments of the family members of evolving DNJ-stems allowed us to identify the apparent mechanism involved, D_H_/V_H_ to DJ_H_ recombination or V_H_ replacement, and thus provide another perspective into our data and results (**Figures 3**-**5**).

D_H_-DJ_H_ segment fusions were first hypothesized in 1982 (40), experimentally verified in 1989 and thereby considered a rare but physiologically legitimate activity adding another combinatorial dimension to the antibody repertoire diversity (11). Since D_H_-DJ_H_ recombination clearly violates the 12-23 rule applying in the process of V(D)J recombination, concerns were raised about a possible aberrant nature of this phenomenon. Further research led to the discovery of hitherto uncharacterized cryptic RSS in the D_H_ genes, confirming the physiological nature of the occasional D_H_-DJ_H_ recombination (10).

Taken together, our data undoubtedly show that all of the described ongoing recombinatory processes, the D_H_/V_H_ to DJ_H_ recombination and V_H_ replacement, drive the clonal evolution in BCP-ALL patients. Moreover, we demonstrate that these mechanisms are almost mutually exclusive and separate patients into clearly delineated groups on different levels:

(i) the D_H_/V_H_-DJ_H_ group was characterized by an overrepresentation of *KMT2A* and *PAX5* aberrations, the presence of a DJ_H_ root, the preferential usage of D_H_-proximal V_H_ genes and the presence of low-abundant DNJ-stem family members, as well as accompanying D_H_-DJ_H_ evolution in *KMT2A* rearranged cases;

(ii) the V_H_ replacement group was (less strongly) associated with the DUX4 and Ph-like molecular subtypes, as well as with D-distal V_H_ genes used by DNJ-stem family members, one or more of which playing the role of abundant mother clonotypes.

The two exceptions to the observation that D_H_-DJ_H_ evolution cases associated with V_H_-DJ_H_ evolution, were two cases belonging to the newly described CDX2/UBTF molecular subtype, which otherwise presented no IGH evolution despite the predominance of a pro-B immunophenotype (26).

Our results point towards D_H_/V_H_-DJ_H_ recombination as an ontogenically earlier-stage and V_H_ replacement as an ontogenically later-stage associated mechanism. In most V_H_-DJ_H_ cases, the DNJ-stem was detectable in the IGH-DJ library, suggesting that V_H_-DJ_H_ is a highly active and ongoing process, resulting in a burst of immature rearrangements. Generally, the biased usage of D_H_/V_H_ genes, particularly V_H_6-1 in V_H_-DJ_H_ recombination and D_H_2-2 in D_H_-DJ_H_ recombination, could be related to the distinctive accessibility of different parts of the V_H_ and D_H_ clusters to the VDJ_H_ recombination machinery as postulated by Tsakou and colleagues (41) and previously shown in mice (42).

The preferential use of V_H_6-1 could at least be related to hampered PAX5 activity. Wild-type PAX5 was reported to cooperate with interleukin receptor 7 to confer locus contraction and subsequent V_H_-DJ_H_ recombination of distal V_H_ segments, whereas it was not required for the preceding D_H_ to J_H_ recombination (43, 44). Consequently, at least the biased usage of V_H_6-1 could be related to the inaccessibility of distal V_H_ segments due to absent or hampered activity of PAX5 and/or related factors.

Our results, which clearly demonstrate a correlation of the molecular subtype with different frequencies and even different mechanisms of clonal evolution, entail clinical consequences. Our selected longitudinal case reports show that MRD quantification, in the form in which it is currently routinely performed, can be affected by the degree of clonal evolution and at worst might lead to false negative MRD results. Thus, the reliability of IGH-based MRD quantification varies depending on the molecular subtype. In particular, patients with the KMT2A subtype, of whom more than 94% are evolving, should be monitored with caution.

Besides the high rates of clonal evolution in *KMT2A-*rearranged BCP-ALL, increasing the susceptibility to errors, studies have shown that IG/TR rearrangements in infant *KMT2A*-rearranged ALL are more likely oligoclonal or even completely absent, which makes any IG/TR based MRD-monitoring less convenient. It has therefore been recommended to preferably employ the *KMT2A* gene fusion for more stable and reliable MRD detection (45, 46).

On the basis of our findings, we suggest the following in order to improve IG/TR-based MRD detection in BCP-ALL: i) follow the DNJ-stem (covering all DNJ-stem family members) and not specific family members; ii) follow the DNJ-stem using both IGH-VJ and IGH-DJ assays; iii) express MRD as the DNJ-stem abundance (the sum of all DNJ-stem family members) in order to avoid underestimation of the MRD level in samples with high degree of clonal evolution; iv) be aware that the classical and still widely adopted real-time quantitative PCR (RQ-PCR) often uses primers and probes designed to track only specific clonal rearrangements (unlike HTS), thus potentially missing family members that are either already present at diagnosis or arise during the course of the disease. Crucially, further testing of the above-suggested approach on large well-defined cohorts is necessary to prove its prognostic strength before it can be routinely applied.

Wrapping up, we will outline a number of future perspectives. First, we herein only studied clonal evolution in the IGH locus, but a study on clonal evolution of T-cell receptors might allow deeper insights - particularly as the TRD locus has shown similar recombinase activity as the IGH locus in patients with V_H_ replacement (47). Second, a recent study has shown that BCP-ALL harboring *TP53* alterations are associated with immature DJ_H_ rearrangements (48). Assuming that *TP53* alterations occur already in the pre-leukemic cell compartment, one should study if these cases show clonal evolution rates similar to *KMT2A*-rearranged cases. Finally, it was recently reported that in childhood BCP-ALL, clonal evolution is not only relevant for reliable MRD detection but also directly correlated to the clinical outcome (33). A similarinvestigation into this critical association of outcome and clonal evolution in adult BCP-ALL would require expanded prospective studies.

## Data availability statement

The data presented in the study are deposited in the European Nucleotide Archive (ENA) at EMBL-EBI, accession number PRJEB59052 (https://www.ebi.ac.uk/ena/browser/view/PRJEB59052).

## Ethics statement

The studies involving human participants were reviewed and approved by Frankfurt Research Ethics Board (188/15F). The patients/participants provided their written informed consent to participate in this study.

## Author contributions

MB, ND, MK and MS designed the research. NG supervised the clinical trial. SS and MS performed immunophenotypic analyses. MK, ND and FD processed, analyzed and interpreted high-throughput sequencing data. ND and KP worked on ARResT/Interrogate. LB, AH and TB analyzed and interpreted RNA-Seq data. FD, ND and MS performed statistical analyses. MB, ND, MS and CB supervised the project. FD, ND, MS, MK and MB drafted the first version of the manuscript. All authors contributed to the article and approved the submitted version.
