# Supplementary material for: Insights into IGH clonal evolution in BCP-ALL: frequency, mechanisms, associations, and diagnostic implications
Source: Front Immunol. 2023 Apr 18;14:1125017. doi: 10.3389/fimmu.2023.1125017 (PMC10151743; doi:10.3389/fimmu.2023.1125017)
Supplement: Supplementary file 1 [file DataSheet_1.docx]

**Supplementary Material**

**S1. Sequencing depth and marker reads**

IGH sequences were analyzed with ARResT/Interrogate. Usable reads, the denominator for percentage abundance calculations, were defined as raw reads with identified VDJ_H_ or DJ_H_ junctions after exclusion of cIT-QC reads, in other words, reads with patient-only rearrangements.

Sequencing depth:

|  | **raw reads** | | | |
| --- | --- | --- | --- | --- |
|  | minimum | maximum | average | median |
| evolving | 3301 | 498354 | 77881 | 33269 |
| stable | 30 | 523937 | 74438 | 34444 |
| no marker | 105 | 525352 | 75571 | 13055 |
|  | **usable reads** | | | |
|  | minimum | maximum | average | median |
| evolving | 1759 | 483034 | 74023 | 30742 |
| stable | 17 | 517378 | 66578 | 33646 |
| no marker | 70 | 516641 | 64870 | 11123 |

Marker reads:

|  | min | max | average | median |
| --- | --- | --- | --- | --- |
| evolving | 57 | 153703 | 9883 | 1722 |
| stable | 35 | 402455 | 14641 | 2494 |

**S2. ARResT/Interrogate: Clonal evolution assessment**

**preliminary steps:**

organize clonotypes by DNJ-stem AND filter for abundance of >=3 and >=0.01% usable reads

**criterion 1:** number of clonotypes under DNJ stem, percentage to total number of clonotypes in sample

IF clonotype percentage >= 5 AND clonotype count >= 10 THEN 'green'

ELSE clonotype percentage >= 1 AND clonotype count >= 10 THEN 'blue'

ELSE clonotype count >= 10 THEN 'blue'

ELSE 'orange'

**criterion 2:** number of clonotypes under DNJ stem

IF clonotype count == 1 THEN 'black'

ELSE clonotype count >= 50 THEN 'green'

ELSE clonotype count >= 10 THEN 'blue'

ELSE clonotype count >= 3 THEN 'orange'

ELSE 'red'

**criterion 3:** clonotype identity/similarity - similar if:

a) by simple/non-aligned string edit distance between nucleotide junction sequences, if edit distance <= 2 and no consecutive mismatches

or b) in the case of VDJ_H_ rearrangements, if D_H_ genes are different, meaning that although rearrangements look different, they might actually be from different DNJ stems

IF clonotype similarity count >= clonotype count THEN 'red'

ELSE clonotype similarity count / clonotype count > 0.5 THEN 'orange'

ELSE 'clear'

**criterion 4:** numbers of different 5' genes and amino acid junction sequence lengths

IF genes == 1 AND lengths >= 1 THEN 'black'

ELSE (genes >= 10 AND lengths >= 10) || (genes + lengths >= 20) THEN 'green'

ELSE genes >= 3 AND lengths >= 3 THEN 'blue'

ELSE genes >= 2 AND lengths >= 2 THEN 'orange'

ELSE 'red'

**criterion 5:** number of N2 nucleotides, increasing the complexity and thus specificity of the DNJ stem

IF N2 length >= 3 THEN 'clear'

ELSE N2 length >= 1 THEN 'orange'

ELSE 'red'

**call by voting with criteria colors:**

IF blacks > 0 THEN 'stable'

ELSE oranges + reds >= blues + greens THEN 'stable'

ELSE blues >= greens THEN 'evolving'

ELSE 'highly evolving'*

*in this study, for simplicity, we did not differentiate between ‘highly evolving’ and ‘evolving

**S3. Longitudinal MRD trajectories in selected BCP-ALL cases**

**Suppl. Figure 1. Visualization of longitudinal MRD trajectories in selected BCP-ALL cases emphasizing pitfalls in MRD detection due to clonal evolution of the marker rearrangement.** (A) Case neg#27, mechanism: V_H_ replacement; (B) Case neg#89, mechanism: V_H_-DJ_H_ recombination; (C) Case neg#50, mechanism: D_H_/V_H_-DJ_H_ recombination; (D) Case neg#61, mechanism: D_H_/V_H_-DJ_H_ recombination. Y-axis: normalized clonotype abundance in % cells. X-axis: annotated timepoints (day +6 samples are peripheral blood, all other samples are bone marrow samples). The kinetics of all family members belonging to the same DNJ-stem under study are visualized. If the DNJ-stem was detectable with both the IGH-VJ and IGH-DJ assay primer tubes, the plots are shown separately. Individual family members are represented as semi-transparent black circles, whose overlap results in less opaque regions. Legend: green/yellow lines = family members of interest as discussed in the main text; vertical background lines at each time point indicate the clonal evolution status of the sample, light red = evolving, light blue = stable; SCT = stem cell transplantation; Blina= blinatumomab; InO= inotuzumab ozogamicin; IND = induction therapy; CONS = consolidation therapy; neg = MRD negative.

For longitudinal case reports with multiple follow-up samples (cohort #5), marker reads (%) were normalized to cells (%) utilizing sample internal spike-in controls (cIT-QC).

**Case Ph-neg #27 with V**_H_ **replacement**

The patient was diagnosed with a common-B ALL and was annotated as Ph-like molecular subtype by transcriptome analysis of a relapse BM sample (Suppl. Figure 1 (A)). The case featured V_H_ replacement as the mechanism driving clonal evolution. At diagnosis, the DNJ-stem was represented by two highly abundant mother clonotypes driving the DNJ-stem’s V_H_ replacement evolution, both generating family members each with their specific V_H_ remnant. Throughout induction II and consolidation phases and until maintenance the MRD remained undetectable (time points not shown). At month 4 of maintenance the MRD increased slowly over 4 additional months (data not shown) and finally reached relapse level (day +718). The initially most abundant family member, mother clonotype 1, (highlighted in green) remained absent in this follow-up sample, however, the mother clonotype 2 seen at diagnosis (highlighted in yellow) persisted throughout maintenance and after one cycle of blinatumomab (day +733). MRD negativity, determined by probing both dominant markers, was only achieved after immediate allogeneic stem cell transplantation (allo-SCT). If only the original most abundant family member, mother clonotype 1, had been followed, it would have led to false MRD negativity at the end of prephase (day +6). Thus, it is important to follow the DNJ-stem in MRD diagnostics and not any single family member, even if they appear most abundant.

**Case Ph-neg #89 with V**_H_**-DJ**_H_ **recombination**

The patient was diagnosed with a pro-B ALL harboring a *KMT2A::AFF1* gene fusion, which was consistently annotated as *KMT2A*-rearranged molecular subtype by transcriptome analysis (Suppl. Figure 1 (B)). The case featured ongoing V_H_-DJ_H_ recombination as the mechanism driving clonal evolution in both BM and PB. The DNJ-stem abundance exceeded the marker threshold (~4.4% cells), while the most abundant family member was detected at a very low level (~0.06% cells, green). After prephase (day +6) all initially present DNJ-stem family members were undetectable in PB, but a new lowly abundant DNJ-stem family member (Suppl. Figure 1 (B), yellow) appeared before the first rituximab dose and was detectable at higher levels (~0.004% of cells) in BM after Induction I (day +21). After three additional doses of rituximab during the subsequent therapy course, this specific family member (Suppl. Figure 1 (B), yellow) ultimately reached marker level (≥1% cells) and the status of first relapse after Consolidation I administered after a prolonged interval (day +142). After allo-SCT (day +184) the family member was still detectable in BM and PB at high abundances. Furthermore, another family member sharing the same DNJ-stem that had been present at diagnosis in both BM and PB, became undetectable in PB at day +6, in BM after Induction I and in the first relapse (day +142), finally reappeared after allo-SCT in PB. In this case we demonstrate that family members sharing the same DNJ-stem might show reciprocal kinetics over time, thus, initial monitoring of the temporarily disappearing family member only would have led to a false negative MRD result and consequently cause a wrong risk stratification after Consolidation I of the patient.

**Case Ph-neg #50 with D**_H_**-DJ**_H_ **and V**_H_**-DJ**_H_ **recombination**

The patient was diagnosed with a c-ALL and annotated as *TCF3::PBX1* molecular subtype by transcriptome analysis (Suppl. Figure 1 (C)). The case featured both D_H_-DJ_H_ and V_H_-DJ_H_ ongoing recombination as the mechanism driving clonal evolution. After an MRD-negative period of 3 months (data not shown), a relapse occurred before reinduction at day +258 after a total of 4 doses of rituximab. At this time point the DNJ-stem had a burst of clonal evolution, while previously only the DNJ-stem in the IGH-VJ library was evolving at diagnosis. In response to the relapse, blinatumomab as secondary immune therapy was administered. At day +8 of the first blinatumomab cycle, while the DNJ-stem in the IGH-VJ library remained evolving, the DNJ-stem in the IGH-DJ library was refractory to therapy and increasing in abundance while its DJ_H_ DNJ-stem family members were decreasing in numbers. This case highlights that IGH clonal evolution can initiate at any time, confirming that evaluation for clonal evolution should not be restricted to initial diagnostic samples. In our detailed analysis we observed that the abundant DJ_H_ clonotype persisting across several follow-up time points was the root for and thus directly enabled both the D_H_-DJ_H_ and V_H_-DJ_H_-related clonal evolution in this patient.

**Case Ph-neg #61 with D**_H_**-DJ**_H_ **and V**_H_**-DJ**_H_ **recombination**

The patient was diagnosed with a pro-B ALL harboring a *KMT2A::AFF1* gene fusion, which was consistently annotated as *KMT2A*-rearranged molecular subtype by transcriptome analysis (Suppl. Figure 1 (D)). The case featured both D_H_-DJ_H_ and V_H_-DJ_H_ ongoing recombination as the mechanism driving clonal evolution. At diagnosis, the evolving DNJ-stem in the IGH-VJ library was above the marker threshold (55% reads, ~15% cells), while the DNJ-stem in the IGH-DJ library was below the marker threshold (1% reads, ~0.3% cells) but also evolving. The DNJ-stem in the IGH-VJ library decreased in abundance after Induction I, while the DNJ-stem in the IGH-DJ library became undetectable. The DNJ-stem in the IGH-VJ library eventually became undetectable after Consolidation I (after 4 doses rituximab), while the DNJ-stem in the IGH-DJ library reappeared indicating refractory disease/early relapse (day +103). One cycle of blinatumomab, given as second-line immune therapy, reduced the MRD burden to the status of low positivity (positive < 1E-04, positive not quantifiable), which was followed by allo-SCT (time points not shown). Two months after allo-SCT a second relapse appeared, which was treated with 1 cycle of inotuzumab ozogamicin (day +248) as third-line immune therapy. The relapse was refractory to the treatment and could be traced by the DNJ-stem in the IGH-VJ library at 0.003% cells, while the DNJ-stem in the IGH-DJ library was undetectable – but in this case it proved critical to call MRD positivity in the pre-SCT sample after Consolidation I (Suppl. Figure 1 (D), red arrows). Consequently, if an evolving DNJ-stem in the IGH-VJ library is also found in the IGH-DJ library sample (and thus considered as rooted), one should also follow the DNJ-stem in the IGH-DJ library for MRD diagnostics even if it does not fulfil marker criteria in the diagnostic sample.
